# Disturbances of Continuous Sleep and Circadian Rhythms Account for Behavioral Difficulties in Children with Autism Spectrum Disorder

**DOI:** 10.3390/jcm9061978

**Published:** 2020-06-24

**Authors:** Enise Yavuz-Kodat, Eve Reynaud, Marie-Maude Geoffray, Nadège Limousin, Patricia Franco, Frédérique Bonnet-Brilhault, Patrice Bourgin, Carmen M. Schroder

**Affiliations:** 1 Centre National de la Recherche Scientifique CNRS UPR 3212, Université de Strasbourg, Institut des Neurosciences Cellulaires et Intégratives, 8 allée du Général Rouvillois, 67000 Strasbourg, France; enise.yavuz@etu.unsitra.fr (E.Y.-K.); pbourgin@unistra.fr (P.B.); schroderc@unistra.fr (C.M.S.); 2 Department of Child and Adolescent Neurodevelopmental Psychiatry, Le Vinatier Hospital, 95 Boulevard Pinel, 69678 Bron CEDEX, France; Marie-Maude.GEOFFRAY@ch-le-vinatier.fr; 3 Health Services and Performance Research (HESPER), Claude Bernard University Lyon 1, 43 Boulevard du 11 Novembre 1918, 69622 Villeurbane CEDEX, France; 4 Department of Neurology and Clinical Neurophysiology, University Hospital Bretonneau, 2 Boulevard Tonnellé, 37044 Tours, France; N.LIMOUSIN-CHAMPFAILLY@chu-tours.fr; 5 Pediatric Sleep, Hôpital Femme-Mère-Enfant, Hospices civils of Lyon, 59 Boulevard Pinel, 69500 Bron, France; patricia.franco@chu-lyon.fr; 6 Lyon Neuroscience Research Center U1028/UMR5292, Claude Bernard University Lyon 1, 43 Boulevard du 11 Novembre 1918, 69622 Villeurbane CEDEX, France; 7 UMR 1253, iBrain, Université de Tours, Inserm, CHRU de Tours, Centre Universitaire de Pédopsychiatrie, 2 Boulevard Tonnellé, 37044 Tours, France; frederique.brilhault@univ-tours.fr; 8 Sleep Disorders Center, International Research Center for ChronoSomnology, Strasbourg University Hospitals, 1 place de l’Hôpital, 67000 Strasbourg, France; 9 Department of Child and Adolescent Psychiatry, Strasbourg University Hospitals & University of Strasbourg Medical School, 67000 Strasbourg, France

**Keywords:** autism, ASD, children, sleep, circadian rhythm, pediatrics

## Abstract

Sleep disorders are among the most common comorbidities in children with Autism Spectrum Disorder (ASD), and subjectively defined sleep disturbances have been related to ASD symptom severity. However, no study has investigated the differential impact of objectively measured sleep and circadian rhythm disturbances on behavioral difficulties in this population. Fifty-two children with ASD aged 3–10 years underwent assessments of sleep and circadian rest–activity rhythms objectively with actigraphy and subjectively with the Children’s Sleep Habits Questionnaire. Behavioral difficulties were assessed using the ABC-C. Group comparison analyses were used to compare sleep and circadian rhythm parameters of children with higher and lower behavioral difficulties and dominance analysis to rank predictors and address multicollinearity. Children with high irritability had a shorter continuous sleep period compared to those with lower irritability (−60 min, *p* = 0.04), as well as those with high stereotypic behaviors compared to children with less stereotypies (−75 min, *p* = 0.006). Objective circadian and sleep disturbances accounted together for, respectively, 17%, 18% and 36% of the variance in social withdrawal, irritability and stereotypic behaviors. The identification of both sleep and circadian rhythm disturbances as explanatory factors for behavioral difficulties warrants their inclusion in the existing behavioral management strategies for children with ASD.

## 1. Introduction

Autism Spectrum Disorder (ASD) is a neurodevelopmental disorder characterized by persistent impairments in social communication and social interaction and the presence of restricted, repetitive patterns of behavior, interests or activities [1]. Its worldwide reported prevalence approaches 1% of the population, and comorbidities are highly prevalent [2,3,4]. Indeed, over 70% of individuals diagnosed with ASD have concurrent somatic or psychiatric conditions such as behavioral disorders, anxiety, gastrointestinal problems, seizures and sleep disorders [5,6,7]. Sleep disorders are among the most common associated disorders in this population, with prevalence rates ranging from approximately 50% to 80% [8,9].

The most common parent-reported sleep problems in children with ASD are related to bedtime resistance, sleep initiation, long periods of nighttime awakening and shortened sleep time [8,10,11]. Studies using objective measures of sleep have observed similar results with increased sleep onset latency, decreased sleep efficiency and increased number and duration of night waking in this population compared to typically developing children [12,13,14,15,16]. Objective sleep was measured either with the gold standard polysomnography or with actigraphy (ACT). The latter is being more and more used to assess sleep in children with ASD, as it is more feasible and allows ecological recordings over longer periods of time, and was recently validated against polysomnography [17].

Alongside studies on sleep itself, a growing number of studies have reported abnormalities of the circadian organization of sleep–wake rhythms in children with ASD. The circadian timing system orchestrates numerous rhythms such as core body temperature, the neuroendocrine system, cardiac function as well as the timing of the sleep–wake cycle. Endogenous melatonin, which is released by the pineal gland at night, plays a crucial role in the regulation of the sleep–wake cycle, acting as a time cue to stabilize and synchronize various circadian rhythms. In a recent systematic review, two main circadian rhythm disorders were reported in children with ASD: a phase delay of sleep periods and an irregular sleep–wake pattern [18]. These circadian rhythm abnormalities in ASD may be linked to mutations in the expression of clock genes [19,20]. Most importantly, a dysregulation in the melatonin rhythm with a decrease in melatonin secretion over the night and globally over the 24-h cycle has been suggested as the main underlying pathophysiological mechanism of circadian rhythm disorders in individuals with ASD [21,22,23,24]. Increasingly, studies monitor the 24-h rest–activity pattern using a wrist-worn actigraphy as a proxy of the circadian sleep–wake rhythm. Sleep–wake rhythms over long periods in children with ASD have rarely been assessed objectively with ACT but were rather carried out in adults with ASD [25,26,27].

In typically developing children, numerous studies have evidenced the association of sleep disturbances with behavioral outcomes (for review, see [28,29]). The majority of these studies have assessed sleep subjectively and only a few studies have investigated circadian rhythms in relation to daytime functioning. In children with ASD, most studies agree that subjective sleep problems are linked to behavioral difficulties [14,16,30,31,32]. However, results in the literature differ with respect to specific subjective sleep problems and their relation to specific behavioral difficulties. Aathira et al. (2017) [33] found an association between poor sleep and overall behavioral difficulties in their study which included 71 children with ASD aged 3–10 years. The differences were particularly important for the symptomatology of being “withdrawn”. Allik et al. (2006) [32] found that parent-reported insomnia was related to hyperactivity in 32 children with Asperger’s Syndrome/High-Functioning Autism with a mean age of 10.8 years. In a sample of 1784 children aged 3–18 years, Goldman et al. (2011) [34] observed contradictory results regarding the association between sleep and hyperactivity, which differed according to the type of behavioral questionnaire used. Subjective sleep disturbances have also been related to the severity of ASD symptoms such as the presence of restricted and repetitive behaviors and difficulties in reciprocal social interactions, as well as irritability, affective disturbances and disruptive behaviors [14,30,31,35,36].

Only a few studies have investigated objectively measured sleep in relation to behavioral difficulties. In a recent study, Bangerter et al. (2020) [37] showed an association between actigraphy-derived sleep efficiency, number of awakenings and hyperactivity and anxiety in children with ASD. However, Anders et al. (2012) [38] found no association between actigraphy-defined sleep problems and behavioral difficulties, including internalizing and externalizing behaviors in young children with ASD. Furthermore, studies using polysomnography in relation to behavioral difficulties are scarce and are usually conducted in children over 10 years on average [12,15,39]. Only one study has investigated the aforementioned relation in children aged 4–10 years [14]. The authors showed a positive correlation between sleep latency and several behavioral problems such as affective problems, anxious/depressed problems and aggressive behaviors.

The literature on the impact of circadian rhythm disorders on daytime functioning in children with ASD is even more scarce and discrepancies remain [22,23,40]. Indeed, Tordjman et al. [22,23] showed a negative correlation between nocturnal urinary melatonin and autism severity, with lower levels of melatonin secretion being associated with lower overall level of verbal language, less imitative social play and more repetitive use of objects; however, other authors did not confirm these findings [40].

To the best of our knowledge, no study has yet objectively investigated the impact of both sleep and circadian rhythm disturbances on behavioral difficulties in children with ASD. Characterizing sleep and circadian rhythms difficulties separately is essential as they have different pathophysiological pathways, as they may have a differential impact on daytime functioning and as they may lead to different treatments. The main objective of this study was to investigate, for the first time, the relationship between subjective and objective measures of sleep and circadian rest–activity rhythm to problem behaviors, and to determine the relative contribution of each in explaining problem behaviors in children with ASD.

## 2. Experimental Section

### 2.1. Participants

Fifty-two children aged 3–10 years were recruited as part of a longitudinal French multicenter clinical research program (University Hospitals of Strasbourg, Lyon and Tours) (Clinical Trial NCT02878499) examining the role of sleep and circadian rhythms disorders in children with ASD. The study complied with the standards of good clinical practices and the Declaration of Helsinki of 1975, as revised in 2008. All procedures contributing to this work have been approved by the regional French Institutional Review Board (Comité de Protection des Personnes “Est IV”, 4 November 2012, Strasbourg, France). Written, signed and informed consent was obtained prior to participation from the parents of participants and assent was obtained from the child when possible.

Inclusion criteria were to be a child aged 3–10 years, with a diagnosis of ASD using the Diagnostic and Statistical Manual of Mental Disorders (DSM IV-TR or DSM-5 criteria) [1]. All participants underwent a clinical diagnostic evaluation by certified practitioners and met criteria for ASD using the Autism Diagnostic Observation Scale cutoff (ADOS [41]), as well as the criteria on all domains of the Autism Diagnostic Interview-Revised (ADI-R [42]). Children were included independently of their degree of developmental delay or intellectual disability associated with ASD. They had to be on no medication or on stable medication 2 months before the inclusion and during the assessment periods. 

Exclusion criteria were ASD associated with known neurogenetic disorders (e.g., associated with fragile X syndrome, Rett syndrome, Down syndrome, Smith–Magenis syndrome, Bourneville tuberous sclerosis, Von Recklinghausen’s disease, cytomegalovirus encephalitis, congenital rubella syndrome and phenylketonuria), epilepsy, comorbid severe physical disability or severe allergy. Finally, participants were not allowed to have had transmeridian travels over two time zones or more, 3 months before the assessments.

### 2.2. Measures

#### 2.2.1. Objective Sleep and Circadian Measures, Using Actigraphy

Actigraphy is a convenient objective and noninvasive method for measuring continuously, in the home environment, both sleep and circadian rest–activity rhythms over long periods of time. Each child was asked to wear an activity monitor with luxmeter (MotionWatch 8^®^, CamNtech Ltd., Fenstanton, Cambridgeshire, UK) for 15 days, on their non-dominant wrist, or on the left wrist by default if the child was not yet lateralized. 

The actigraph is an electronic device containing a piezo-electric accelerometer that measures the intensity, amount and duration of physical movement in all directions. Actigraphic activity is measured in counts defined as the amplitude of the signal produced by its accelerometer, with the number of counts being proportional to the intensity of the movement. The accelerometer samples movement amplitude 32 times per second. Its peak of intensity is defined for each second as the highest amplitude across the 32 records, and peak intensity values were summed into an epoch of 1 min.

##### Sleep Analysis

Actigraphy data were scored automatically for sleep/wake using the Actiwatch Activity and Sleep Analysis 7^®^ software algorithm, version 7.23. As our previous actigraphy validation study in children with ASD indicated that the low sensitivity-threshold setting shows the best fit when compared to polysomnography (PSG) [17], this setting is thus reported here. The low sensitivity-threshold is defined as a threshold level of 80 counts, i.e., an activity score of 80 counts or more during an epoch of 1 min is necessary for that epoch to be scored as wake.

Sleep parameters obtained by the sleep analysis were: Total Sleep Time (TST), Sleep Latency (SL), Wake After Sleep Onset (WASO) and Sleep Efficiency (SE). TST was defined as the time between sleep onset and sleep offset minus the time of WASO, and SL was defined as the time between bedtime and sleep onset. WASO was defined as the number of minutes scored as wake between sleep onset and sleep offset. SE was defined as the ratio of TST to the time in bed (i.e., time from bedtime to get up time).

In addition to classic sleep parameters provided by sleep analysis software, we assessed the longest continuous sleep episode (LSE). LSE is defined as the longest duration of uninterrupted sleep and has recently been identified as a clinically meaningful sleep parameter in a study population of children and adolescents with ASD [43,44,45,46,47]. Previous studies have measured LSE subjectively using sleep diaries, whereas it was objectively measured using actigraphy in the present study. As such, LSE was defined here as the longest uninterrupted sleep episode or interrupted by less than five consecutive minutes of scored wake on ACT. This modification was necessary due to the brief episodes of sleep that may be identified incorrectly as a wake episode [17]. The definition of LSE was based on a comparison of ACT and PSG in this sample (unpublished data), which identified a tolerance margin of 5 min as the most fitting one, when compared to LSE measured by PSG.

In parallel to the actigraphy recording, parents were requested to fill a paper-based sleep diary and to indicate a set of information on their child’s sleep including bedtime (the time the child is in bed and ready to sleep) in order to calculate sleep latency. Sleep diary data were used to reduce actigraphy artifacts, including the removal of the device (e.g., bathing, showering and swimming pool) and any reason for an atypical night of sleep (e.g., sickness, sleepover, late evening event, etc.).

##### Circadian Measures

Non-Parametric Circadian Rhythm Analyses (NPCRA; MotionWare 1.2.31, Cambridge Neurotechnology, Fenstanton, Cambridgeshire, UK) were performed using rest–activity data to assess three actigraphy-derived circadian rhythm parameters: interdaily stability (IS), intradaily variability (IV) and relative amplitude (RA). Algorithms that determine these variables were described by Van Someren et al. (1999) [48]. To perform NPCRA, at least 4 continuous 24-h days of monitoring were required (whereas only nighttime recording of at least 4 days were required for sleep analysis). If the actigraph device was removed for more than half an hour and up to 3 h a day, the activity level of this missing period was replaced by the average of the mean activity level of the day before and the day after this missing period. If more than 3 h of recording were missing in a day, that day was not considered in the NPCRA. As parents kept a sleep diary during the period of monitoring, it was possible to handle missing data and/or artifacts with parents’ comments (e.g., child removed the actigraph device while in the swimming pool).

The parameter of IS gives an indication on the day-to-day stability of the rest–activity rhythm. IS values range between 0, for no stability other than Gaussian noise, to 1, indicating that the rest–activity pattern is repeated perfectly every single day. A higher IS value indicates a more stable rhythm.

IV provides information on the variability of the rhythm within a day and quantifies the degree of fragmentation of the behavioral rhythm. In other words, IV indicates the frequency and extent of transitions between rest and activity. Its value ranges between 0, indicating that transitions between rest and activity within a day are tightly consolidated, to 2, which means that the fragmentation of transitions between rest and activity is random. 

RA is an estimate of the strength or robustness of the circadian rhythm. It is defined as the normalized difference between the most active 10-h period and least active 5-h period in a 24-h pattern. Higher values indicate greater amplitude with values ranging between 0 and 1.

The midpoint of sleep (MSF), another actigraphy-derived circadian parameter, was also computed [49]. It is defined as the midpoint between sleep onset and sleep end with the calculation as follow: time of sleep onset + TST/2. This measure is indicative of the circadian preference of a child, i.e., its chronotype. In our study sample of children aged 3–10 years at inclusion, we did not correct for sleep debt accumulated during work or school days (no significant differences between sleep on school days and weekends).

#### 2.2.2. Subjective Sleep Measures

##### The Children’s Sleep Habits Questionnaire (CSHQ)

The CSHQ is a parent-rated questionnaire comprised of 45 items, 33 of which contribute to the scoring, designed to screen for sleep problems in children aged 4–10 years [50]. Parents were asked to complete the questionnaire based on their child’s sleep habits over the last typical week. Each scored question is rated on a three-point scale as occurring “usually” (i.e., 5–7 times per week), “sometimes” (i.e., 2–4 times per week) or “rarely” (i.e., never or once a week). To date, the CSHQ has generally been used to assess the severity of sleep problems or to categorize children as “good sleepers” or “poor sleepers” based on the CSHQ total score, with a clinically sensitive cut-off score established to ≥41 for the identification of probable sleep problems [50]. In their initial study, Owens and colleagues (2000) demonstrated a very poor to acceptable internal consistency of the eight subscales (Cronbach’s alphas: 0.36–0.70), although test–retest reliability was acceptable (correlations values: 0.62–0.79). This initial study did not assess the factor structure and, thereafter, only a few studies have investigated its psychometric properties in typically developing children, with none of them supporting the original eight subscales structure of the CSHQ [51]. The psychometric examination of the CSHQ factor structure allows to establish a profile of specific types of sleep problems rather than assessing the overall severity of sleep problems. To date, despite its widespread use in children with ASD, only 3 studies have investigated the factor structure of the CSHQ in this population. Johnson and colleagues [52] proposed a revised five-factor structure while Katz and colleagues [53] suggested a four-factor structure, in children with ASD aged 2–10 years and 4–10 years, respectively. We based the scoring of the CSHQ on a recent study investigating the factor structure of the questionnaire in a population corresponding closely to our population of interest: ASD children aged 4–5 years [54]. In this ASD-adapted CSHQ, the questionnaire is comprised of 5 subscales: Bedtime Routine (BTR), Sleep Onset and Duration (SO&D), Night Wakening (NW), Sleep Disordered Breathing (SDB) and Morning Wakening (MoW).

#### 2.2.3. Behavioral Difficulties

##### Aberrant Behavior Checklist-Community (ABC-C)

The ABC-C is a 58-item parent-rated questionnaire that was initially developed for the assessment of a range of disruptive behaviors in people with intellectual and developmental disabilities [55]. The ABC-C was shown to have sound psychometric properties with high internal consistency across the subscales (mean alpha = 0.91), excellent test–retest reliability (mean *r* = 0.98) and acceptable inter-rater reliability (mean *r* = 0.63). Its use has been validated in children with ASD [56].

Each question is rated by parents/caregivers on a four-point scale from 0 (“not a problem”) to 3 (“the problem is severe in degree”). The ABC-C is comprised of five factors: ABC-I, Irritability, Agitation, Crying (15 items); ABC-II, Lethargy and social withdrawal (16 items); ABC-III, Stereotypic Behavior (7 items); ABC-IV, Hyperactivity, Noncompliance (16 items); and ABC-V, Inappropriate Speech (4 items). The latter was not considered in this study because many young children with ASD in our study had not acquired sufficient levels of speech. Higher scores indicate more severe behavioral difficulties. Since the number of items differs between ABC-C factors, the scores were reported as a percentage to ease comparison (i.e., (raw score × 100/maximum score of each ABC-C factor)).

Recent studies have reported nonlinear associations between sleep and daytime functioning [57,58]. Thus, ABC-C subscales were dichotomized individually according to their median value, to study the difference in sleep and circadian rest–activity rhythm between subjects with higher and lower behavioral difficulties, allowing for an easier clinical interpretation. When participants scored equal to or above the median on an ABC-C factor, they were referred as the group with “higher problem behavior” and as the group with “lower problem behavior” when participants scored under the median on that ABC-C factor.

#### 2.2.4. Adaptive Behaviors

To describe the level of adaptive functioning of the study sample, the Vineland Adaptive Behavior Scales (VABS [59]) was administered. The VABS is a semi-structured interview conducted with parents to assess a range of adaptive behaviors such as communication, socialization, daily living skills and motor skills. Equivalent ages for each domain were reported here. 

### 2.3. Data Analysis and Statistics

Two sets of analyses were conducted in the present study. First, we investigated the association between each of the sleep and circadian variables with the 4 ABC-C factors. To compare sleep and circadian measures between the group with higher and the group with lower problem behavior for each ABC-C factor, independent sample *t* tests, Wilcoxon Mann–Whitney or Welch’s *t*-tests were performed, according to data distribution and homogeneity of variance.

Secondly, we carried out dominance analysis (DA) to determine, among objective sleep and circadian exposures, those most strongly associated with behavioral difficulties. Dominance analysis allows both addressing multicollinearity between predictors and ranking predictors according to the relative importance of their contribution in explaining the variance of the outcome. Addressing multicollinearity was essential as sleep and circadian measures are strongly correlated; thus, classic linear regression was not considered. 

DA consists in exhaustive series of pairwise comparisons of the predictors, considering all possible subset regression models with and without the other predictors. For example, when considering 3 predictors (X1, X2 and X3), 7 models are created: three models including one predictor alone, three including 2 predictors each (X1 and X2), (X1 and X3) and (X2 and X3)) and a last model including all three predictors. The additional contribution of a given predictor is measured by the increase in explained variance that results from adding that predictor to the regression model. Thus, the additional contributions of X1 are the increases in the proportion of variance accounted for when X1 is added to each subset of the remaining predictors (i.e., the null subset, X2, X3, X2 and X3). One predictor is said to completely dominate another if its additional contribution to each of the subset models is greater than that of the other predictor [60,61,62]. 

Three DA were tested: in the first model, named the “sleep model”, all objective sleep predictors were entered (TST, WASO, SL and LSE) except sleep efficiency as its calculation is dependent on the other sleep predictors. In the second DA, named the “circadian model”, all circadian predictors were entered: IS, IV, RA and MSF. In the last DA, the “circadian and sleep model”, both objective sleep and circadian predictors were entered: TST, WASO, SL, LSE, IS, IV, RA and MSF. The coefficient of determination, *R*^2^, for each model (*R*^2^ total) and for the dominant predictor within each model are reported. 

Statistical analysis was performed using R version 1.2.5001. The calc.yhat package was used to perform dominance analysis [61]. Statistical significance was set at *p* value < 0.05.

## 3. Results

### 3.1. Study Participants

The sample included 52 children comprised of 41 boys (79%) and 11 girls, with a mean age of 5.39 years ± 1.50 (SD) and an age range of 2.75–9.57 years. Descriptive characteristics of the study participants are set out in Table 1. As measures were not always interpretable (e.g., incomplete questionnaires, insufficient number of days of ACT, etc.), the number of participants for which the measure was available is specified. The average developmental delay (i.e., the difference between chronological age and developmental age) in adaptive behaviors according to the Vineland Adaptive Behaviors Scale subdomains ranged 1.85–2.93 years and was especially important in the subdomains of communication and socialization.

### 3.2. Comparison of Sleep and Circadian Rhythm Measures between Behavioral Difficulty Groups

**Figure 1:**
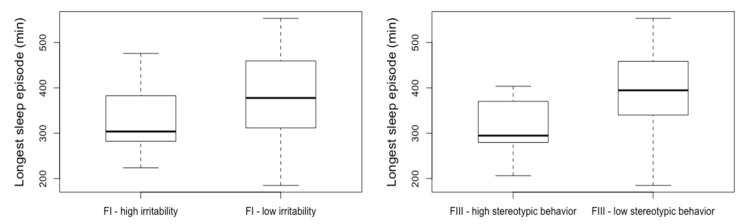
Longest continuous sleep episode as a function of problem behaviors in children with ASD. FI—irritability, Factor I, Irritability on the Aberrant behavior checklist–Community (ABC-C). FIII—stereotypic behavior, Factor III, stereotypic behavior on the Aberrant behavior checklist–Community. Low, group with an ABC-C score below the median, calculated separately by factor; High, group with an ABC-C score equal or above the median, calculated separately by factor.

Table 2 reports the differences in sleep and circadian measures between the groups with higher problem behaviors and the groups with lower problem behavior, separately for the four ABC-C factors. Within objective sleep measures, the longest sleep episode showed the highest difference between groups with low or high levels of behavioral disturbances. On average, the higher irritability group slept continuously 60 min less than the lower irritability group (*p* = 0.04). The group with higher stereotypic behaviors slept continuously 75 min less than the lower stereotypic behaviors group (*p* = 0.006) (see Figure 1). In other words, the less continuously the children slept, the more irritability and stereotypic behavior they displayed. No differences were observed regarding the objective circadian rest–activity rhythm measures.

When comparing subjective sleep measures between the groups with higher problem behaviors to the groups with lower problem behaviors, a significant difference was found on several ASD-adapted CSHQ subscales, as well as on the total CSHQ, scored according to the original scoring. The group with higher irritability differed significantly from the group with lower irritability on bedtime routine (*p* = 0.033). A difference between groups was also observed for social withdrawal on sleep onset and duration (*p* = 0.034). The group with higher hyperactivity did not differ from the group with lower hyperactivity but a tendency was found for bedtime routine (*p* = 0.069). The group with higher social withdrawal significantly differed from the group with lower social withdrawal on the CSHQ total score (*p* = 0.048).

### 3.3. Dominance Analyses

The dominance analyses (DA) enabled to determine separately for the four ABC-C factors, the importance of the contribution of objective sleep and circadian predictors and to identify which predictor is most instrumental. The results are reported in Table 3, with the first analysis including as predictors the objective sleep measures (TST, WASO, LSE and SL), the second including the objective circadian measures (IS, IV, RA and MSF) and the third including both. Total R² indicates the coefficient of determination of the model (i.e., the proportion of the variance of ABC-C factors explained by the predictors combined), a dominant predictor is reported with its unique variance when fulfilling complete dominance requirement (cf. Methods Section, paragraph 2.3. Data Analysis and Statistics).

**Figure 2:**
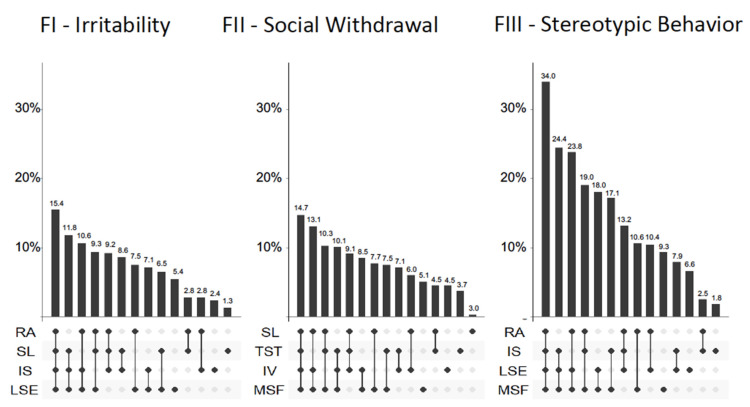
Differential effect of sleep and circadian rhythm parameters on problem behaviors in children with ASD. Percentage of explained variance of ABC-C factors across dominance analysis models. Each column represents a model; the filled dots indicate which variables are included. For each factor, the four variables with the highest unique variance were retained, and the corresponding models included 1–4 variables. Models representing less than 1% are not shown. FI–irritability, Factor I, Irritability on the Aberrant Behavior Checklist–Community; FII–social withdrawal, Factor II, social withdrawal on the Aberrant Behavior Checklist–Community; FIII–stereotypic behavior, Factor III, stereotypic behavior on the Aberrant Behavior Checklist–Community. Sleep variables: LSE (longest sleep episode), TST (total sleep time) and SL (sleep latency). Circadian variables: RA (relative amplitude), IS (interdaily stability), IV (intradaily variability) and MSF (midpoint of sleep).

In the sleep and circadian model, the eight objective predictors accounted for 17.7% of the variance in irritability, and the longest sleep episode (LSE) contributed 6.1% of unique variance (see Figure 2). The same model accounted for 16.7% of the variance in social withdrawal, with the midpoint of sleep (MSF) as a complete dominant factor with 4.7% of unique variance. The variance in stereotypy was explained by the combination of sleep and circadian parameter accounting for 35% of the variance. Although no factor reached individual complete dominance, MSF and LSE dominated the other predictors. The MSF contributed approximately 18% of unique variance in stereotypic behavior and LSE accounted for 14.3% of unique variance in stereotypic behavior. For the last ABC-C factor, hyperactivity, the sleep and circadian model accounted for only 0.7% of the variance in hyperactivity and relative amplitude contributed 0.4% of unique variance.

## 4. Discussion

This is the first study to undertake an objective and subjective assessment of sleep and circadian rest–activity rhythm in association with behavioral difficulties in young children with ASD. Longer continuous sleep in children with ASD was associated with less behavioral difficulties during the day. Indeed, the duration of the longest uninterrupted sleep episode, as measured objectively by actigraphy, was 60 min shorter in the group with higher irritability than the group with lower irritability, and 75 min shorter in the group with higher stereotypy than the group with lower stereotypy (see Figure 1). A 60–75-min period is equivalent to at least one complete sleep cycle (REM sleep and non-REM sleep) in children (50–80 min, depending on age). Both REM sleep and non-REM sleep are related to child growth and memory consolidation [63,64,65], and there is a general consensus that consolidated sleep throughout a whole night is optimal for the plasticity changes needed for learning and memory consolidation in children with ASD [66]. This study thus corroborates the interest of the longest sleep episode as a marker of sleep quality related to daytime outcomes in children with ASD. In accordance with these results, a previous study found that a clinically meaningful improvement in the duration of the longest sleep episode, after a 13-week treatment of pediatric prolonged-release melatonin, led to a significant improvement in daytime behavioral problems in a population of 125 ASD children aged 2–17.5 years [46]. Additionally, this study found that the longest sleep episode was also associated with improvement in quality of life in parents. Together, these findings support the importance of continuous nocturnal sleep for daytime behavioral functioning and highlight the importance of this sleep measure when assessing sleep in children with ASD, as well as the repercussion of sleep on overall quality of life of their families.

Using dominance analyses, we observed that objective circadian and objective sleep disturbances accounted together for, respectively, 17%, 18% and 36% of the variance in social withdrawal, irritability and stereotypic behaviors. The longest sleep episode and the midpoint of sleep were the most dominant predictors to explain the variance of these problem behaviors (see Figure 2).

Although it is essential to provide objective sleep and circadian measures, subjective data have their own value, as some sleep disorders diagnoses, in particular insomnia, are solely defined through subjective criteria. In the present study, children with higher irritability had more difficulties in the following subjective measures: bedtime routine, while children with higher social withdrawal showed insufficient sleep duration and higher bedtime resistance. Moreover, the group with higher social withdrawal had significantly more severe sleep problems, based on the CSHQ total score. The group with higher hyperactivity did not differ from the group with lower hyperactivity on subjective measures, but a tendency was found for bedtime routine.

Comparative literature is scarce, as the majority of studies using objective assessment of sleep have only focused on characterizing sleep in children with ASD. Only a few studies have investigated actigraphy-derived sleep measures in relation to behavioral difficulties. A recent study has shown an association between actigraphy-derived sleep efficiency, number of awakenings and hyperactivity and anxiety in 144 children with ASD aged 14.6 on average [37]. In contrast, apart from the longest sleep episode mentioned above, other objective sleep measures in our study were not significantly associated to behavioral difficulties in our population of very young children with ASD. Our findings are in accordance with a previous study which has found no association between actigraphy-defined sleep problems and behavioral difficulties, including internalizing and externalizing behaviors, in 68 children with ASD with a mean age of 3.9 years [38]. Discrepancies among studies may thus be attributed to the age of the study population, with links between objective sleep measures and behavior being more difficult to establish in very young children with ASD. Contradictory results may also be explained by different actigraphy-related methods for the estimation of sleep parameters. Indeed, the algorithm that determines sleep or wake and the definition of sleep parameters differ. For example, in the study of Bangerter et al. (2020) [37], sleep efficiency was defined as the percentage of minutes of sleep within the interval between sleep start and sleep end, whereas it was calculated as the actual sleep time divided by the time in bed in the present study. This difference may bias toward a higher estimation of sleep efficiency in their study compared to ours. Furthermore, we attempted to compare the results with other objective sleep measures such as polysomnography, but very few studies have investigated sleep in relation to behavioral difficulties and the majority have been conducted in children over 10 years on average and have rather focused on the relation to autism severity [12,39,67]. Only one study investigated the aforementioned relation in children aged 4–10 years [14]. The authors showed a positive correlation between sleep latency and several behavioral problems such as affective problems, anxious/depressed problems and aggressive behaviors. Altogether, these findings suggest that further studies in children with ASD relating objective sleep measure to behavioral difficulties are required in order to clearly establish the precise association between these variables for each age group.

As for subjective sleep, a growing literature has investigated its impact on behavioral difficulties. Globally, studies agree that sleep problems are linked to behavioral difficulties in children with ASD [14,16,30,31,32]. However, the results in the literature differ with respect to specific sleep problems and their relation to specific behavioral difficulties. In accordance with our findings, Aathira et al. (2017) [33] also found an association between poor sleep, defined with a cutoff score on the CSHQ, and overall behavioral difficulties, measured by the total score on the Child Behavior Checklist (CBCL), in their study on 71 children with ASD aged 3–10 years. The differences were particularly important for the symptomatology of being “withdrawn”. Similarly, the association between sleep and irritability has been established in previous studies [31,68]. Indeed, Allik et al. (2006) [32] found that parent-reported insomnia was related to hyperactivity rated on the Strength and Difficulties Questionnaire (SDQ) in 32 children with Asperger’s Syndrome/High Functioning Autism with a mean age of 10.8 years. In another study, in a sample of 1784 children aged 3–18 years, Goldman et al. (2011) [34] established a significant difference in hyperactivity between ASD-good sleepers and ASD-poor sleepers on the Parental Concern Questionnaire but not on the CBCL externalizing behaviors [34]. Unlike these studies, we did not find any association between hyperactivity and subjective sleep [30,32,34,69]. The discrepancy between our results and the literature can be partly explained by the difference in age and the method used across studies in order to characterize either sleep or hyperactivity. Indeed, as can be seen in the study by Goldman et al. (2011), the type of questionnaire used to assess hyperactivity can explain diverging results. Moreover, the aforementioned studies examined the relationship between sleep and hyperactivity in children who are older than those in our study sample, and the effects of sleep problems are likely to differ according to age. It is also possible that, in young children with ASD, hyperactivity may be exacerbated but that sleep does not play a preponderant role in this particular behavior in the studied age and that any added effects of sleep may only be visible in studies with larger sample sizes. We tested this hypothesis by conducting separate analyses in preschool-aged children (<6 years, *n* = 33) and school-aged children (≥6 years, *n* = 19). In schooled-aged children, the group with higher hyperactivity had a significantly lower sleep efficiency compared to the group with lower hyperactivity (*p* = 0.028), while this association was not found in preschool-aged children (*p* = 0.12). 

As the methods to assess and analyze subjective sleep are different across studies, precise comparison is hindered. Indeed, the majority of studies used the original scoring of the CSHQ while the present study used the ASD-adapted scoring. Since changes in sleep difficulties occur over time, from preschoolers to middle childhood, a recent study included children with ASD aged 4–5 years old and provided a novel five-factor model [54]. In the present study, the mean age of the sample was 5.39 years old which is similar to that of Zaidman-Zait et al. (2020) [54]. Thus, this novel five-factor model was retained for our analyses.

Results on actigraphy-derived circadian measures did not show any difference in children with higher behavioral difficulties compared to children with lower behavioral difficulties. While it is possible that there is no difference to be found, another explanation for these results may be that actigraphy-derived non-parametric circadian rhythm analyses (NCPRA) may be an inadequate proxy of circadian rhythms in our population of very young children with ASD and significant sleep disturbances. As mentioned above, NCPRA give three actigraphy-derived circadian rhythm parameters: IS, IV and RA. These circadian rest–activity rhythm measures represent a proxy of the endogenous rhythm; they do not reflect it directly. While this approximation may be adequate in the population in which they were conceived, they are not necessarily fitting in all population. Indeed, they were initially investigated in adults with Alzheimer’s disease [48] and were later used in other populations such as in Parkinson’s disease, bipolar disorder or in healthy infants [70,71,72]. There are different possible reasons for which the NPCRA might not correctly identify the circadian rhythm of young children. Children’s sleep–wake cycles depend on their parents rather than their internal rhythms, many still nap and thus display a biphasic circadian rhythm and they present more movement during sleep. While actigraphy-derived measures of sleep have been validated against its gold standard, polysomnography [17], this is yet to be done for NPCRA measures, which have not yet been validated against a gold standard measurement of circadian rhythms such as melatonin or core body temperature.

Although between-group analyses did not show any difference in the midpoint of sleep, a proxy of circadian preference or chronotype [49], dominance analysis (DA) enabled to highlight the importance of these circadian measures in explaining behavioral difficulties when studied together with sleep measures. Indeed, the midpoint of sleep completely dominated all other predictors in explaining social withdrawal, accounting uniquely for around 5% of the variance. DA also emphasized the role of the chronotype measures studied together in explaining stereotypy and social withdrawal accounting, respectively, for 18.7% and 4.7% of their variance. These results can be paralleled to those of Sun et al. (2018) [73] who demonstrated, in 12-month-old typically developing infants, the implication of the circadian rest–activity rhythm in the development of social learning. Clinically significant social withdrawal has been reported as a common behavior in ASD children in previous studies [74]. Altogether these findings suggest that circadian preference, or chronotype, may explain in part the social disability that is described in children with ASD, possibly linked to either genetic factors or to rhythm desynchronization. Furthermore, the relation of sleep and circadian abnormalities to stereotypic behaviors may be explained by a common physiopathological pathway. Indeed, serotonin, which is the precursor of the circadian neurohormone melatonin, has been found to be involved in various functions such as sleep, circadian rhythms, affective regulation and stereotypic behaviors [75,76]. Increased whole-blood serotonin levels and decreased plasma melatonin have previously been reported in patients with ASD [20,77]. The link between serotonin levels and stereotypic behaviors is further evidenced by the efficacy of medications that inhibit serotonin transport [78] and an exacerbation of these behaviors after a pharmacological depletion of tryptophan which leads to reduced serotonin synthesis [79].

A particular strength of the present study is the objective assessment of both sleep and circadian rhythms with actigraphy, while the majority of studies addressing the same topic have assessed sleep mainly subjectively. Furthermore, only a small body of research has evaluated circadian rhythms objectively in relation to daytime behavior and none related to behavioral difficulties. Our study sample included ASD children aged 3–10 years across the spectrum, with and without associated intellectual disability. This allows generalizing our results to the overall population of ASD children and not only to high-functioning ASD children, which is often the case in the literature. Even though our study sample is larger than most studies assessing sleep objectively in relation to daytime behavior, it may not possess sufficient statistical power to show between-group differences. Indeed, a clinically significant mean difference of 28 min in WASO was observed between the groups with lower versus higher stereotypic behaviors, but statistical significance was not reached. A further limitation of the present study is the use of a single measure to characterize children’s daytime behavior. Indeed, it would have been valuable to assess behaviors with questionnaires that are specific of ASD symptoms such as, the Social responsiveness Scale [80] or the Repetitive Behavior Scale [81], or using observational data. In the present study, we conducted a cross-sectional analysis, which does not allow establishing causality and addressing the issue of temporality. To do so, a longitudinal analysis is required. The present analyses were run on the baseline measures of an ongoing longitudinal research project. The longitudinal analyses will allow us to address temporality in the future. The objective evaluation of the circadian rest–activity rhythm is both a strength and a limitation. Indeed, actigraphy enables to noninvasively record circadian rest–activity rhythms, thereby providing an estimation of the child’s ecological sleep–wake rhythm over long periods of time, but it remains a proxy of the circadian timing system. Therefore, future studies should address this limitation and use gold standard phase markers of the circadian rhythm such as 24 h melatonin profile or core body temperature. One possible way to better characterize the circadian rhythm with actigraphy would be to validate the NPCRA against gold standard measures first, and then to establish normative values across different age groups in both typically developing children and in children with ASD. Only recently, a few studies have attempted to address this research gap [82,83,84]. However, only one study investigated the NPCRA, involving solely 24-h recordings, in children ages 4–11 years [83]. Further work on this topic using longer follow-up periods is therefore needed. As mentioned above, in the present study, we used actigraphy rather than polysomnography as actigraphy has recently been validated against polysomnography in children with ASD [17] and because actigraphy allows for a continuous record of sleep during a week or more, in the home environment, unlike polysomnography, which is often conducted in a sleep laboratory for 1–2 days. In addition to that, we performed dominance analyses, which allow addressing multicollinearity between predictors and ranking predictors according to the relative importance of their contribution in explaining the variance of the outcome unlike classic regression analysis.

## 5. Conclusions

In the present study, we showed that problem behaviors were strongly accounted for by both sleep and circadian rhythm disturbances. In particular, the longest continuous sleep episode is a novel clinically meaningful sleep parameter to consider, especially in children with severe sleep disorders. Our results suggest that the longest sleep episode may be a choice candidate particularly when examining sleep in its relation to behavioral phenotype in children with ASD.

The identification of sleep and circadian rhythm disturbances as explanatory factors for a range of behavioral difficulties warrants their systematic assessment and inclusion in the existing behavioral management strategies. This would enable future development of a comprehensive intervention program in young children with ASD that may improve responses to currently available behavioral management programs.

## Figures and Tables

**Table 1:** Descriptive characteristics of the study participants.

	Mean (SD)	Range
VABS subdomains (*n* = 47) (developmental age)		
Communication (years)	2.7 (1.4)	(0.8–5.6)
Socialization (years)	2.4 (1.3)	(0.9–6.5)
Daily living skills (years)	3.1 (1.8)	(0.9–11.1)
Motor skills (years)	3.4 (1.2)	(1.4–6.2)
ABC-C subscales (*n* = 52)		
I: Irritability, agitation, crying	43.3 (24.0)	(0.0–93.3)
II: Lethargy, social withdrawal	27.4 (15.0)	(4.2–72.9)
III: Stereotypic behavior	27.9 (19.3)	(0.0–76.0)
IV: Hyperactivity, Non-compliance	50.5 (23.5)	(2.0–98.0)
CSHQ total score (*n* = 50)	51.6 (9.4)	(34–78)
ASD-adapted CSHQ (*n* = 50)		
Bedtime routine	6.2 (2.4)	(4–12)
Sleep onset and duration	10.4 (2.7)	(6–16)
Night waking	7.8 (1.9)	(6–12)
Sleep disordered breathing	3.5 (1.3)	(3–9)
Morning wakening	6.7 (2.1)	(4–12)
Actigraphy sleep measures (*n* = 40)		
TST (min)TST(hours)	503.9 (52.5)	(372.0–603.1)
	8.40 (52.5)	(6.2–10.1)
WASO (min)	51.8 (23.0)	(21.9–126.1)
SE (%)	82.6 (6.0)	(65.9–91.0)
SL (min)	36.8 (23.6)	(4.0–94.1)
LSE (min)	354.0 (90.0)	(184.8–553.1)
LSE (hours)	5.9 (1.5)	(3.1–9.2)
Actigraphy circadian measures (*n* = 39)		
IS	0.59 (0.11)	(0.33–0.78)
IV	0.65 (0.09)	(0.44–0.88)
RA	0.93 (0.05)	(0.79–0.98)
MSF (hh:mm)	02:07 (01:06)	(23:46–04:19)

VABS, Vineland Adaptive Behavior Scales; ABC-C, Aberrant behavior checklist-community; ABC-I, Factor I Irritability; ABC-II, Factor II social withdrawal; ABC-III, Factor III stereotypic behavior; ABC-IV, Factor IV Hyperactivity; ABC-total, Total score on the ABC-C; CSHQ, Children’s sleep habits questionnaire; BR, bedtime resistance; SOD, sleep onset delay; SD, sleep duration; NW, night waking; Para, parasomnia; SDB, sleep disordered breathing; DS, daytime sleepiness; CSHQ total; Total score on the CSHQ; ASD-adapted CSHQ, ASD-adapted Children’s sleep habits questionnaire; BTR, Bedtime Routine; SO&D, Sleep Onset and Duration; NW, Night Wakening; SDB, Sleep Disordered Breathing; MoW, Morning Wakening; TST, Total Sleep Time; WASO, Wake After Sleep Onset; SE, Sleep Efficiency; SL, Sleep Latency; LSE, longest Sleep Episode; IS, Interdaily Stability; IV, Intradaily Variability; RA, Relative Amplitude; MSF, Midpoint of Sleep.

**Table 2:** Sleep and circadian rhythm measures by ABC-C factor group.

	I-Irritability Mean (SD)		II-Social Withdrawal Mean (SD)		III-Stereotypy Mean (SD)		IV-Hyperactivity Mean (SD)	
	Low	High	Low	High	Low	High	Low	High
**Objective sleep (ACT)**								
TST (min)	509.6 (63.3)	498.2 (39.8)	506.8 (59.5)	501.2 (46.7)	512.2 (54.5)	495.6 (50.5)	503.4 (62.4)	504.6 (34.6)
SE (%)	82.8 (7.2)	82.3 (4.8)	82.4(6.8)	82.7(5.4)	84.0 (5.5)	81.1(6.3)	82.2(7.1)	83.1 (4.1)
SL (min)	38.3 (23.4)	35.3 (24.4)	39.4 (28.1)	34.4 (19.1)	36.2(25.6)	37.4 (22.2)	36.9 (23.0)	36.7 (25.3)
WASO (min)	48.4 (26.1)	55.2 (19.3)	50.0 (21.23)	53.5 (24.8)	56.3 (26.8)	84.0 (5.6)	52.3 (28.0)	51.1 (13.2)
LSE (min)	383.1 (101.6) *	324.8 (67.1) *	370.3 (101.9)	339.1 (77.2)	391.7 (90.5) **	316.2 (73.7) **	354.1 (100.3)	353.7 (75.0)
**Circadian rhythm (ACT)**								
IS	0.62 (0.09)	0.58 (0.12)	0.61 (0.11)	0.58 (0.10)	0.62 (0.10)	0.57 (0.11)	0.59 (0.11)	0.60 (0.10)
IV	0.65 (0.08)	0.65 (0.10)	0.64 (0.09)	0.66 (0.10)	0.65 (0.08)	0.65 (0.11)	0.65 (0.09)	0.65 (0.10)
RA	0.93 (0.05)	0.92 (0.04)	0.93 (0.04)	0.92 (0.05)	0.93 (0.04)	0.92 (0.05)	0.93 (0.05)	0.92 (0.05)
MSF (hh:mm)	01:57 (01:15)	02:17 (00:54)	01:59 (01:13)	02:14 (00:59)	02:18 (00:57)	01:56 (01:13)	02:07 (01:05)	02:08 (01:09)
**Subjective sleep (ASD-adapted CSHQ)**								
BTR	5.4 (2.0) *	7.0 (2.6) *	5.7 (2.5)	6.8 (2.2)	6.0 (2.7)	6.4 (2.1)	5.6 (2.0)	6.9 (2.7)
SO&D	9.8 (2.4)	11.1 (2.8)	9.7 (2.9) *	11.2 (2.2) *	10.1 (2.9)	10.8 (2.4)	10.2 (2.6)	10.8 (2.8)
NW	7.6 (1.8)	8.0 (2.0)	7.3 (1.6)	8.3 (2.1)	7.4 (1.6)	8.2 (2.1)	7.9 (1.9)	7.7 (1.9)
SDB	3.6 (1.5)	3.4 (1.1)	3.5 (1.4)	3.6 (1.3)	3.6 (1.5)	3.4 (1.1)	3.8 (1.7)	3.2 (0.7)
MoW	6.8 (2.1)	6.6 (2.3)	6.4 (2.1)	6.9 (2.2)	6.6 (2.2)	6.7 (2.1)	6.7 (2.2)	6.6 (2.2)
CSHQ total	49.3 (7.6)	53.4 (10.4)	48.9 (8.8) *	54.2 (9.2) *	50.2 (9.4)	52.8 (9.2)	50.8 (9.3)	52.2 (9.4)

* *p* < 0.05, ** *p* < 0.01. Low, group with an ABC-C score below the median, calculated separately by factor; High, group with an ABC-C score equal or above the median, calculated separately by factor; ABC-C, Aberrant behavior checklist-community ABC-I, Factor I Irritability; ABC-II, Factor II social withdrawal; ABC-III, Factor III stereotypic behavior; ABC-IV, Factor IV Hyperactivity; ABC-total, Total score on the ABC-C; ACT, actigraphy; TST, Total Sleep Time; SE, Sleep Efficiency; SL, Sleep Latency; WASO, Wake After Sleep Onset; LSE, longest Sleep Episode; IS, Interdaily Stability; IV, Intradaily Variability; RA, Relative Amplitude; MSF, Midpoint of Sleep (average and standard deviation are ported in hh:mm). ASD-adapted Children’s sleep habits questionnaire subdomains: BTR, Bedtime Routine; SO&D, Sleep Onset and Duration; NW, Night Wakening; SDB, Sleep Disordered Breathing; MoW, Morning Wakening; CSHQ total, Children’s sleep habits questionnaire total score.

**Table 3:** Dominance analysis of sleep, circadian predictors and disruptive behaviors.

		I-Irritability	II-Social Withdrawal	III-Stereotypy	IV-Hyperactivity
**Sleep**	*R*^2^ total	7.0%	6.0%	7.0%	0.8%
	Dominant predictor (*R*^2^)	LSE (2.5%)	TST (4.1%)	LSE (4.3%)	SL (0,3%)
**Circadian**	*R*^2^ total	4.5%	9.8%	18.4%	0.3%
	Dominant predictor (*R*^2^)	IS (1.7%)	MSF (5.2%)	MSF (15.8%)	RA (0.3%)
**Sleep and Circadian**	*R*^2^ total	17.7%	16.7%	35.9%	0.7%
	Dominant predictor (*R*^2^)	LSE (6.1%)	MSF (4.7%)	LSE & MSF *	RA (0.4%)

* Complete dominance between LSE and MSF cannot be established. MSF accounted uniquely for 18.2% and LSE for 14.3% for the stereotypic behaviors factor. IV, intradaily variability; RA, relative amplitude; MSF, mid-point of sleep; ABC-I, Factor I Irritability; ABC-II, Factor II social withdrawal; ABC-III, Factor III stereotypic behavior; ABC-IV, Factor IV Hyperactivity; TST, total sleep time; LSE, longest sleep episode.
